# Rate of Belowground Carbon Allocation Differs with Successional Habit of Two Afromontane Trees

**DOI:** 10.1371/journal.pone.0045540

**Published:** 2012-09-26

**Authors:** Olga Shibistova, Yonas Yohannes, Jens Boy, Andreas Richter, Birgit Wild, Margarethe Watzka, Georg Guggenberger

**Affiliations:** 1 Institute of Soil Science, Leibniz Universität Hannover, Hannover, Germany; 2 VN Sukachev Institute of Forest, Siberian Branch of Russian Academy of Sciences, Krasnoyarsk, Russia; 3 Ethiopian Institute for Agricultural Research, Addis Ababa, Ethiopia; 4 Department of Chemical Ecology and Ecosystem Research, University of Vienna, Vienna, Austria; DOE Pacific Northwest National Laboratory, United States of America

## Abstract

**Background:**

Anthropogenic disturbance of old-growth tropical forests increases the abundance of early successional tree species at the cost of late successional ones. Quantifying differences in terms of carbon allocation and the proportion of recently fixed carbon in soil CO_2_ efflux is crucial for addressing the carbon footprint of creeping degradation.

**Methodology:**

We compared the carbon allocation pattern of the late successional gymnosperm *Podocarpus falcatus* (Thunb.) Mirb. and the early successional (gap filling) angiosperm *Croton macrostachyus* Hochst. es Del. in an Ethiopian Afromontane forest by whole tree ^13^CO_2_ pulse labeling. Over a one-year period we monitored the temporal resolution of the label in the foliage, the phloem sap, the arbuscular mycorrhiza, and in soil-derived CO_2_. Further, we quantified the overall losses of assimilated ^13^C with soil CO_2_ efflux.

**Principal Findings:**

^13^C in leaves of *C. macrostachyus* declined more rapidly with a larger size of a fast pool (64% *vs*. 50% of the assimilated carbon), having a shorter mean residence time (14 h *vs*. 55 h) as in leaves of *P. falcatus*. Phloem sap velocity was about 4 times higher for *C. macrostachyus*. Likewise, the label appeared earlier in the arbuscular mycorrhiza of *C. macrostachyus* and in the soil CO_2_ efflux as in case of *P. falcatus* (24 h *vs*. 72 h). Within one year soil CO_2_ efflux amounted to a loss of 32% of assimilated carbon for the gap filling tree and to 15% for the late successional one.

**Conclusions:**

Our results showed clear differences in carbon allocation patterns between tree species, although we caution that this experiment was unreplicated. A shift in tree species composition of tropical montane forests (e.g., by degradation) accelerates carbon allocation belowground and increases respiratory carbon losses by the autotrophic community. If ongoing disturbance keeps early successional species in dominance, the larger allocation to fast cycling compartments may deplete soil organic carbon in the long run.

## Introduction

The residence time of the assimilated carbon in an ecosystem is a function of its allocation in the plant-soil system [1]. While carbon allocated in fast-cycling tissues and compounds is quickly returned to atmosphere by respiration, carbon incorporated into structural compounds of the plant or transformed to slow-cycling soil organic matter has a much longer lifetime and, by this, determines the long-term carbon sequestration [2], [3]. Consequently, modification in the carbon allocation pattern by compositional and structural changes of the forest vegetation affects carbon pools and turnover and may influence the carbon balance at ecosystem scale [4], [5], [6].

Isotope tracer techniques give the opportunity to follow carbon fluxes in the plant-soil system *in situ*. During the last few years good progress has been made with ^13^C and ^14^C pulse labeling experiments of whole trees to measure the carbon allocation along with the contribution of recent photosynthates to soil CO_2_ efflux, e.g., see reviews of Brüggemann *et al*. [7] and Epron *et al*. [8]. Studies in boreal [9], [10], [11] and temperate [12], [13], [14] forests provided convincing evidence that soil respiration is closely linked to photosynthesis and that the contribution of recently assimilated carbon to soil CO_2_ efflux is large. The transfer of assimilated carbon belowground may account for up to 25–63% of gross primary production [15]. Once belowground, it becomes rapidly available for metabolic processes in the autotrophic continuum, i.e., roots and soil biota closely associated with roots [3], [16], [17]. Recently gained carbon can be rapidly transferred belowground, influencing soil CO_2_ efflux rates on short-time scales (from hours to days) [1], [3], [18], [19]. The rate of carbon transfer depends both on environmental factors as well as on the tree species. Transfer rates are reported to slow down with decreasing temperature and soil moisture [14], [20], [21], tentatively due to changes in the turgor pressure gradients between the source and sink organs [8]. Pulse labeling experiments also showed broadleaved tree species characterized by much higher carbon transfer rates than coniferous species [8], [21]. However, as emphasized by Epron *et al*. [8], hitherto pulse labeling studies have been restricted to temperate and boreal forests, while trees from tropical ecosystems have not yet been assessed. Given the importance of tropical forests in the climate change debate, this is a serious shortcoming, since prolonged vegetation period and higher biomass production suggest pronounced differences to temperate forests.

Though tropical ecosystems, and particularly African forests, have been recognized as a globally relevant carbon sink [22], [23], [24], there is still great uncertainty on magnitude and variability of the African carbon stocks and fluxes [25], [26]. Controversially, evaluations of African carbon stocks have found the carbon balance of African ecosystems to range from a sink of about 0.3 Pg C yr^−1^ to a small source, mainly depending on whether the rate of deforestation and forest degradation had been taken into account [23], [27]. For sub-Saharan Africa, Ciais *et al.* [27] estimated the CO_2_ emission induced by forest degradation and deforestation to be about 0.24 Pg C yr^−1^. In addition, scant information on the effects of anthropogenic pressure on representative African forest ecosystems makes the regional estimates and biogeochemical models of carbon cycle highly uncertain [23], [24]. In addition, there is little information concerning the effects of anthropogenic pressure on representative African forest ecosystems, which makes the regional estimates and biogeochemical models of carbon cycle highly uncertain [23], [24].

In contrast to tropical lowlands, Afromontane ecosystems are often highly productive in agricultural terms and thus densely populated. Consequently, there deforestation as well as forest degradation is much more pronounced than in the lowlands. As a consequence, deforestation as well as forest degradation is much more pronounced than in the lowland areas. The often fertile soils in east African mountains areas also have larger organic carbon stocks than their counterparts in the lowlands [28]. This makes them an important potential source of greenhouse gases due to deforestation and forest degradation [29]. The latter aspect is also important as the Afromontane forest belt covers a relatively large area extending at altitudes above 2000 m from Sierra Leone in the west to Somalia in the east and from the Sudan Republic in the north to the Cape Peninsula in the south [30]. Among them the Ethiopian highlands contribute to more than 50% by area of the tropical Afromontane vegetation [31]. Ethiopia is representative for the ongoing processes of forest degradation as a result of anthropogenic disturbance such as timber extraction, firewood collection, and in particular forest grazing. In contrast to deforestation, where one type of vegetation is replaced by another with easily recognizable effects on carbon stocks and fluxes, the impact of forest degradation on carbon cycling is considerably more subtle. The Afromontane forests in central and southern Ethiopia have been described as Podocarpus mixed forests characterized by a mixture of evergreen and deciduous tree species with dominance of *Podocarpus falcatus* (Thunb.) Mirb. (Podocarpaceae) and *Croton macrostachyus* Hochst. es Del. (Euphorbiaceae) in the upper canopy [32]. These two coexisting tree species represent two different functional types [33]. While gymnosperm coniferous *P. falcatus* is a late successional shade tolerant tree, the angiosperm *C. macrostachyus* is an early successional light demanding tree (i.e., gap filler). In Ethiopia as in whole Eastern Africa *P. falcatus* is among the tree species that are locally most threatened by illegal cutting and encroachment [34]. These activities create gaps that favor the abundance of early successional tree species like *C. macrostachyus* and cause problems in natural regeneration of *P. falcatus* if occurring too frequently [35], [36]. Along with the drastic decline of *P. falcatus*, *C. macrostachyus* appears to become the most abundant indigenous tree species of Ethiopian highlands [37].

To assess the direction and magnitude of the impact of forest degradation on carbon cycling, the physiological traits of trees have to be taken into consideration [38], [39]. According to Leuschner *et al*. [40], photosynthetic capacity and maximum leaf conductance seem to be clearly different between gap fillers and late-successional tree species with minor or no overlap between the two groups, although considerable interspecific variation exists. Typical gap fillers are fast growing angiosperms, such as *C. macrostachyus*. The usually faster growth of angiosperms as compared to gymnosperms (e.g., podocarps) [41] is the result of the evolutionary gained physiological features, including higher stomata conductance and higher specific leaf area and hence higher photosynthetic capacity, rapid accumulation and cycling of nutrients, and low investment of acquired carbon in wood [42], [43], [44], [45]. In general, gymnosperm podocarps have low photosynthetic rates per unit leaf mass compared to aniosperms [46], and thus low leaf-level nutrient productivity [47]. On the other hand, tropical podocarps have longer lifetimes than tropical angiosperms [48]. A few comparative studies between *P. falcatus* and *C. macrostachyus* report higher rates of photosynthesis and transpiration, larger specific leaf area and a higher metabolic activity for the latter [49], [50], [51].

The degraded Munessa-Shashemene forest in southern Ethiopia is a typical Afromontane forest where anthropogenic disturbance causes higher abundance of the gap filler *C. macrostachyus* at the cost of the late successional *P. falcatus* [35], [36]. The forest provides an excellent opportunity to study *in situ* differences in the carbon allocation by these two coexisting tree species. For this purpose we exposed individual trees *P. falcatus C.* and *macrostachyus* to a stable carbon isotope tracer in order (i) to evaluate the timing of the recently fixed carbon allocation belowground and its respiratory release as CO_2_ by the autotrophic continuum in soil and (ii) to quantify the fraction of newly assimilated carbon lost by soil respiration underneath the tree species. We hypothesize that the velocity of recently assimilated carbon translocation from the tree canopy to soil CO_2_ efflux is faster in case of the gap filling angiosperm *C. macrostachyus*. In addition, we propose that compared with the gymnosperm *P. falcatus*, more carbon is allocated belowground to fuel the autotrophic continuum and is released into the atmosphere as soil CO_2_ efflux.

## Materials and Methods

### Site Description

The study was conducted in the Munessa-Shashemene forest, which that is located at the eastern escarpment of the southern Main Ethiopian Rift Valley, about 250 km south of Addis Ababa (7°26′ N, and 38°52′ E). The climate is sub-humid with a mean annual temperature of about 15°C; and average annual rainfall amounts to 1150 mm [52]. The rainfall pattern has been referred to as bimodal with a minor rainy season from March to May and a major rainy season from July to September [53]. However, meteorological monitoring during the last 9 years revealed that the short dry season in between the minor and the major rainy seasons actually diminished [52]. Site conditions are homogenous [54], with soils developed from volcanic parent material and are rich in clay minerals and iron oxides [53]. According to the World Reference Base of Soil Resources [55], the soils are classified as Mollic Nitisols. There is a mosaic of rudimentary natural forest and forest plantations, which ranges from about 2.000 to 2.700 m a.s.l. However, the natural forest is strongly degraded by grazing and illegal logging. These disturbances favour the early successional *C. macrostachyus*, concentrating primarily at canopy gaps, at cost of late successional species such as *P. falcatus* and *Prunus africana* (Hook. F.) Kalkman.

### Experimental Setup

We established the study plot at an elevation of 2300 m a.s.l. in a typical patch of the degraded natural forest. Dominant canopy species are *C. macrostachyus* (143 trees ha^−1^) and *P. falcatus* (73 trees ha^−1^), and tree species with less abundance include *P. africana, Syzygium guineense* (Wild.) DC., *Celtis africana* Burm. f. and *Pouteria adolfi-friederici* (Engl.) [56].

In July 2008, one pair (experimental and control) of each of *C. macrostachyus* and *P. falcatus* trees were selected at a distance of *c.* 100 m. The trees in pairs were similar in height but differed in stem diameter and foliage mass (Table 1). The projected areas of the canopies of experimental and control trees varied between 12.7 m^2^ and 16.7 m^2^. We assumed these areas to contain the main part of the roots of each considered tree [57]. About six months prior starting the experiment, the roots were trenched around the corresponding areas to 50 cm depth. A net was also installed underneath the canopies to prevent the potential input of labeled leaves onto the soil.

**Table 1:** Main characteristics of the experimental trees.

Characteristic	*Croton macrostachyus*	*Podocarpus falcatus*
Functional group	Gymnospserm	Angiosperm
Life strategy	Early successional	Late successional
Shade tolerance	Light demanding	Shade tolerant
Leaf habit	Facultative shedding, deciduous	Evergreen, coniferous
Leaf life span [51]	Often <1 year, depending on weather conditions	>2 years
Leaf flush [51]	During moist periods	Throughout the whole year with maxima during moist periods
Mycorrhiza type [79]	Arbuscular mycorrhiza	Arbuscular mycorrhiza
Height, m	5.1*; 5.6†	6.2*, 5.6†
DBH, cm	4.5*, 4.1†	11.5*, 9.4†
Foliage mass, g_DW_	780*	4910*

* labeled tree;

† control tree.

Five permanent PVC collars (20 cm diameter) were randomly installed on the forest floor under the canopy of each candidate tree for subsequent measurements of soil CO_2_ efflux and gas sampling. Within the collars, the ground vegetation was gently removed to avoid its contribution to soil CO_2_ efflux.

### Pulse Labeling

Short-term ^13^CO_2_ pulse labeling of *C. macrostachyus* and *P. falcatus* trees was carried out on two consecutive cloudless days (on November, 12 and November, 14, 2008) on the offset of the main rainy season. For the whole tree ^13^C labeling in the remote field area, we used a crown labeling chamber approach as suggested by Simard *et al*. [58], which isolates the soil surface from the chamber headspace. The crown labeling chamber prevented the diffusion of ^13^CO_2_ to soil pores and back to the atmosphere as well as avoided the labeling of understory vegetation. This all increased the accuracy of ^13^CO_2_ efflux measurements under the target trees [13]. We constructed rectangular wooden chambers made of eucalyptus poles around the individual trees about one week before the experiment took place. The chamber volumes were 58.4 m^3^ and 71.1 m^3^ for *C. macrostachyus* and *P. falcatus*, respectively. Within 30 min before the labeling event, a greenhouse plastic cover (UVA Clear, Ginegar Plastics Products Ltd, Israel: 120 micron, 88% light transmission in PAR**)** was pulled over the frame and tightly sealed. The chamber was also sealed with the plastic film from the bottom side at 1.60 m distance from the forest floor. Five electric fans (12V, 0.21A) per chamber circulated the air during labeling, and CO_2_ concentration, air temperature, and relative humidity were monitored continuously using a LI-8100 infrared gas analyzer (LI-COR Inc., Lincoln, NE, USA). A flask with isotopically enriched Na_2_^13^CO_3_ solution (99 atom% ^13^C; Campro Scientific, Berlin, Germany) was placed inside the chamber. Fifteen minutes after the chamber was sealed, and the decline of the CO_2_ concentration inside the chamber indicated carbon dioxide uptake at a reasonable rate, ^13^C-labeled CO_2_ was generated by injecting diluted sulfuric acid into Na_2_^13^CO_3_, and 12.5 mM of ^13^CO_2_ per 1 m^3^ was added to each chamber. The labeling duration was 85 min and 105 min for *C. macrostachyus* and *P. falcatus*, respectively. Thereafter, the plastic cover was removed and the chamber opened (defined as sampling time 0).

Environmental conditions were comparable for both labeling experiments. During the labeling period the air temperature inside the *C. macrostachyus* and *P. falcatus* chambers increased to a maximum of 29.4°C and 27.3°C, respectively, being *c.* 5°C higher than the ambient temperature. Relative air humidity increased from 58% to 79% within the *C. macrostachyus* chamber and from 56% to 82% within the *P. falcatus* chamber, when the ambient air humidity ranged from 58 to 67% and 56 to 69% during the labeling period of the two consecutive days. Photosynthetically active radiation (PAR) within the chambers averaged 1070±317 and 990±400 µmol m^−2^ s^−1^ for *C. macrostachyus* and *P. falcatus* during labeling, respectively.

### Sample Collection

Leaves, phloem sap, soil CO_2_ efflux, and the 0–10 cm soil depth increment were sampled over a one year period at 1, 2, 3, 4, 8, 16, 32, 64, 120, and 365 days after the labeling. In addition, within the first 24 hours after labeling, leaves and phloem sap were sampled in 4 hour intervals, while soil CO_2_ efflux was collected 12 hours after the labeling.

Leaves were randomly sampled from 4 opposite directions from the top, middle and bottom part of the trees crown, both for labeled and control trees. *Croton macrostachyus* lost part of its leaves at the end of a dry period in February 2009. In the following moist period, new leaves have been produced and thereafter leaf samples were represented by a mixture of mature and young leaves. All foliage samples were dried for 48 h at 65°C and ground by a ball mill. Phloem soluble sugars were extracted according to the phloem exudation method described by Gessler *et al*. [59]. Briefly, pieces of phloem tissues (about 1×1 cm) were removed with a scalpel from the stems at 1.3 and 0.5 m height and were immediately transferred to glass vials, containing 2 ml of 15 mM polyphosphate buffer (Sigma, München, Germany). After 5 hours of extraction the supernatant was decanted and kept frozen.

Soil cores were taken from 0–10 cm depth. Within four hours after the soil samples were taken, they were sieved <2 mm, and the ‘adhering soil method’ [60] was employed to separate the rhizosphere soil from the bulk soil. Immediately thereafter the soil samples were frozen until analysis of the neutral lipid fatty acid (NLFA) 16∶1ω5, a marker for arbuscular mycorrhiza (AM) [61].

Soil CO_2_ efflux was measured at each of the PVC collars placed underneath the canopy of the study trees. A 8100–103 Survey Chamber (LI-COR Inc., Lincoln, NE) was tightly fitted on the collars, and soil CO_2_ efflux was recorded by a LI-8100 infrared gas analyzer [54]. The LI-8100 system was also used to collect gas samples for analyzing the ^13^C signature of the CO_2_ emitted from soil. For this, a T-fitting with septum was installed between the analyzer unit and the survey chamber. At each sampling time, gas samples were taken from all 5 PVC collars at the control and labeled plots, respectively. Every collar 15 ml of air was sampled with a 20-ml syringe equipped with 6-cm long needle and injected into a previously evacuated 12 ml glass vial (Exetainer, Labco Ltd, High Wycombe, UK) to produce an overpressure, thus preventing a contamination with ambient air during storage. In addition, at each sampling time, five air samples were taken at the labeled and control plots at approximately 100 ppm steps of increasing CO_2_ concentration inside the survey chamber to establish Keeling plots [62].

### Stable Carbon Isotope Analysis

The stable carbon isotope composition was analyzed for leaves, water soluble phloem sugars, NLFA 16∶1ω5 extracted from adhering and bulk soil, and soil-derived CO_2_.

To determine the stable carbon isotope ratio in leaves, *c.* 1 mg of finely ground material was analyzed on a Thermo Finnigan MAT DELTA^plus^ Advantage isotope ratio mass spectrometer (Thermo Electron Corporation, Waltham, USA) coupled to an Euro EA 1110 C/N analyzer (EuroVector SpA, Milan, Italy) or, alternatively, on an Elementar IsoPrime 100 IRMS (IsoPrime Ltd., Cheadle Hulme, UK) coupled to an Elementar vario MICRO cube EA C/N analyzer (Elementar Analysensysteme GmbH, Hanau, Germany).

For compound-specific isotope analysis of the phloem sugars, an HPLC system (Dionex Corporation, Sunnyvale, CA, USA) was coupled to a Finnigan Delta V Advantage Mass Spectrometer by a Finnigan LC IsoLink Interface. Briefly, sample compounds were first separated by the HPLC system. Then the individual compounds were oxidized to CO_2_ in the Finnigan LC IsoLink Interface, excess O_2_ was removed oxidizing elemental copper, and the O_2_-free gas stream was transferred to the mass spectrometer for stable isotope analysis. Standards were referenced with EA/IRMS (Euro EA 1110 CN analyzer coupled to a Finnigan MAT DELTA^plus^ IRMS) as pure chemicals before preparing the solutions. Details of the analytical procedure including the correction of the HPLC/IRMS data can be obtained in [63]. Based on the individual concentrations and isotope composition of the sugars glucose, fructose and sucrose a weighted average of the isotope composition of the mono- and disaccharides in the phloem sap was calculated.

Lipid extraction followed the method of Frostegård et al. [64]. At first step the NLFA were extracted with a chloroform-methanol citrate buffer (1∶2:0,8, v/v/v) from 1g of adhering and 1.5g of bulk soil, followed by solid phase extraction with silica SPE columns (Varian Bond Elute LRC-Si; Agilent Technologies, Santa Clara, CA) to obtain the neutral lipids by chloroform extraction. Thereafter, the neutral lipids were subjected to mild alkaline methanolysis to obtain the NLFA methyl esters. The derivatized NLFA were separated by gas chromatography (Agilent 7890A; Agilent Technologies, Santa Clara, CA), oxidized to CO_2_ by an Isoprime GC V Interface, and measured for stable carbon isotopes on an Elementar IsoPrime 100 IRMS. The ^13^C enrichment of the 16∶1ω5 NLFA was corrected for the carbon added in the methanolysis step of the fatty acid analysis procedure.

The stable carbon isotopic ratio of soil-respired CO_2_ was analyzed by continuous-flow isotope-ratio mass spectrometry on a Thermo Finnigan Delta V Advantage Mass Spectrometer (Thermo Fisher Scientific, Waltham, MA, USA) coupled to a Finnigan GasBench.

### Calculations

Isotope values are expressed in δ notation (‰), relative to the Vienna Pee Dee Belemnite (VPDB) standard. All δ^13^C values (‰) were converted to the absolute isotope ratio (^13^C/^12^C) of the sample (*R_sample_*):
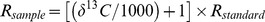
(1)where *R_standard_* is the standard value for isotope ratio of VPDB. The fractional abundance (*A*) of ^13^C relative to *^12^C+^13^C* was then related to *R* by equation:



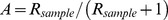
(2)The excess ^13^C in the foliage of the labeled trees (*^13^C_FL_*, g) was calculated by multiplication of the difference in the fractional abundances in leaves of the labeled trees and the control trees with the leaf biomass carbon of the labeled trees as following:
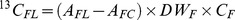
(3)where *A_FL_* and *A_FC_* is ^13^C fractional abundance of leaf samples from labeled and control trees respectively, *DW_F_* is total dry foliage biomass of labeled trees in g and *C_F_* is foliage carbon concentration of the labeled trees (437 mg g^−1^ for *C. macrostachyus* and 462 mg g^−1^ for *P. falcatus*).

The isotopic signature of the soil CO_2_ efflux collected with the closed chamber was estimated by calculation of the intercept *a* of the Keeling plot relationship.
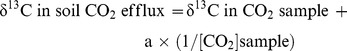
(4)at each sampling time [62]. Keeling plots with *R^2^*<0.9 were discarded. No correction was carried out for physical isotopic fractionation and mixing processes of δ^13^C in soil-respired CO_2_. However, the consequences of these non unambiguous data should be negligible as the label effect on soil respiration is nearly two orders of magnitude larger than the isotopic effect of diffusion. To calculate the excess ^13^C in soil CO_2_ efflux under the labeled trees as compared to that under the control trees at each sampling point, the fractional abundance A (equation 2) and the measured soil CO_2_ efflux rate were used to calculate the amount of ^13^C evolved from soil (*^13^C_SRL_*, g ^13^C m^−2^ h^−1^) under the labeled and control trees:
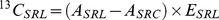
(5)where 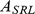 and 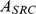 is ^13^C fractional abundance of soil CO_2_ efflux under labeled and control trees, respectively; and 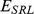 is the soil CO_2_ efflux rate under labeled trees (mg m^−2^ h^−1^) recorded during the correspondent sampling time. To minimize the temporal variability in isotopic ratio values due to changes in environmental parameters we used the difference between the values of the individual collars from labeled and control trees.

The cumulative excess of ^13^C in soil CO_2_ efflux between two points in time (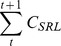, g ^13^C m^−2^ time period^−1^) was calculated according to:
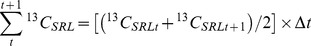
(6)where *^13^C_SRLt_* and *^13^C_SRLt+1_* is the excess of ^13^C in soil CO_2_ efflux (g ^13^C m^−2 ^h^−1^) at two consecutive points in time, and *Δt* defines the time interval (h) between the two points in time. The cumulative excess ^13^C in soil CO_2_ efflux during the 365 days chasing period 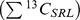
was obtained by summarizing the cumulative excess of ^13^C for the different time intervals.

The mean residence time (MRT) and half-life of the label in foliage and the soil ^13^CO_2_ efflux were calculated by fitting exponential functions to the cumulative excess of ^13^C in the foliage and of ^13^C in soil CO_2_ efflux in the one-year time course. Mean residence times *(1/K)* and half lives *(ln(2)/K)* are expressed in h.

The rate of ^13^C assimilation by the trees could not be analyzed directly, because non-dispersive infrared gas analyzer underestimates the true values of CO_2_ concentration in ^13^CO_2_ enriched atmosphere due to the shift in the absorption spectrum of ^13^CO_2_ relative to that of ^12^CO_2_ [65], [66]. Therefore, the ^13^C assimilation was estimated by two independent indirect ways. Approach 1 was based on the known atom percent of ^12^CO_2_ and ^13^CO_2_ in the chamber directly after adding the defined amount of label [67]. Thus the measured decline of CO_2_ inside the chamber following the labeling was the sum of the real ^12^CO_2_ uptake and the apparent ^13^CO_2_ uptake. Considering an isotopic discrimination of ^13^C against ^12^C during assimilation of 0.973 [68], the real ^12^CO_2_ uptake and the apparent ^13^CO_2_ uptake during the labeling period could be distinguished. The real ^13^C concentration was then estimated by multiplying the apparent ^13^CO_2_ uptake by a factor of 4.62, which was obtained by comparing the measured increase in the CO_2_ concentration in the chamber and the expected increase based on the amount of ^13^CO_2_ added.

Approach 2 was based on the measured ^13^C enrichment in leaves immediately after the labeling (time 0) related to the foliage biomass of the labeled trees, which was estimated by an allometric approach [57], [69]. Fifteen *C. macrostachyus* and 15 *P. falcatus* trees of comparable tree height and diameter at breast height as the study trees were selected. From each tree, 15 randomly selected branches were cut and the basal branch diameter of each branch was measured. The leaves from each branch were harvested and dried (65°C for 48 h). The dry weight of the foliage was related to the basal branch diameter of the corresponding branch by using a single exponential function *y = a exp^bx^*, with *R^2^* of 0.70 and 0.87 for *C. macrostachyus* and *P. falcatus*, respectively, with *P*<0.0001.

## Results

### ^13^C Assimilation and Recovery in Foliage

**Figure 1:**
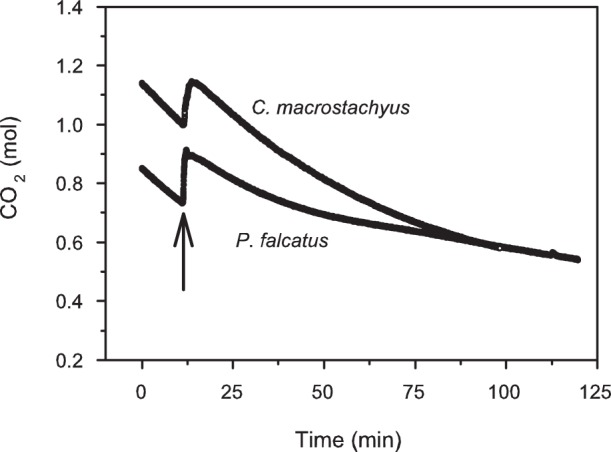
Change in the apparent amount of CO_2_ (mol) in chambers of *Croton macrostachyus* and *Podocarpus falcatus* during the ^13^CO_2_-labeling period. The decline in the amount of CO_2_ reflects the predomination of photosynthesis over leaf and stem respiration. The arrow shows the release of 12.3 mmol ^13^CO_2_ m^−3^ chamber volume.

Carbon dioxide concentration declined inside the chambers during the labeling, indicating a photosynthetic uptake by the trees (Fig. 1). The calculated rate of carbon uptake of *C. macrostachyus* was higher (12.7±2.2 µmol m^−2^ chamber basal area s^−1^) in comparison to that of *P. falcatus* (8.5±3.1 µmol m^−2^ chamber basal area s^−1^). Overall, *C. macrostachyus* and *P. falcatus* assimilated *c*. 0.4 and 0.5 mol, or 5.2 and 6.5 g ^13^C, respectively.

**Figure 2:**
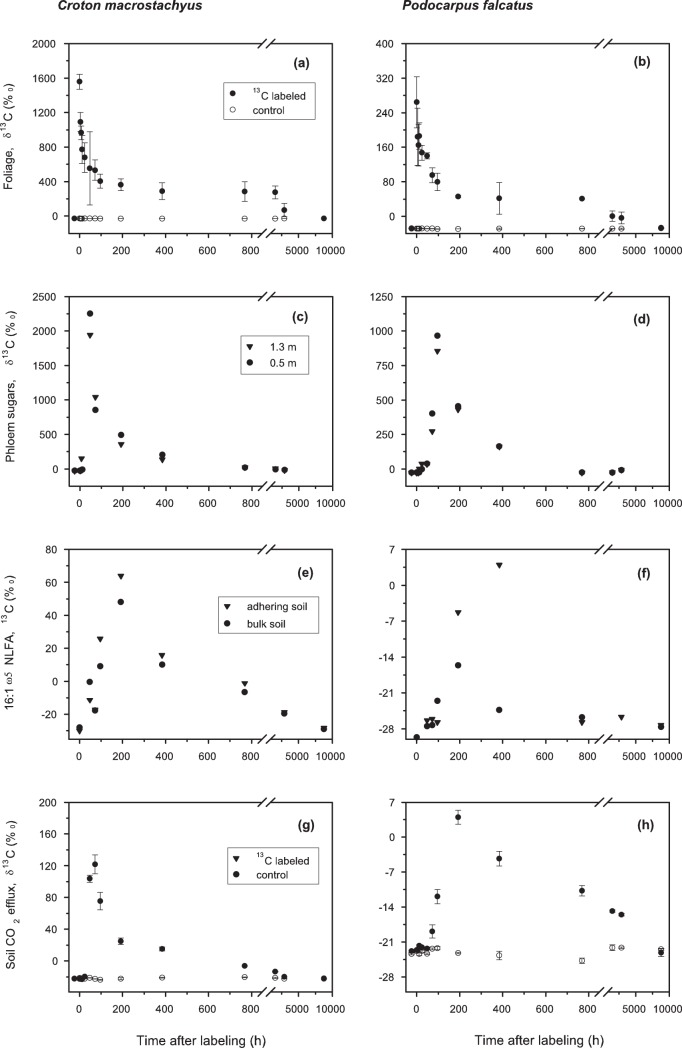
Time course of the ^13^C label in plant-soil compartments of *Croton macrostachyus* and *Podocarpus falcatus* during the one year chasing period. Shown are **(a)** leaves of *C. macrostachyus*, **(b)** leaves of *P. falcatus*, **(c)** weighed sum of soluble mono and disaccharides in tree phloem sap at 1.3 m and 0.5 m above ground of *C. macrostachyus*, **(d)** weighed sum of soluble mono and disaccharides in tree phloem sap at 1.3 m and 0.5 m above ground of *P. falcatus*, **(e)** 16∶1ω5 NLFA in adhering and bulk soil under *C. macrostachyus*, **(f)** 16∶1ω5 NLFA in adhering and bulk soil under *P. falcatus*, **(g)** soil CO_2_ efflux under *C. macrostachyus*, **(h)** soil CO_2_ efflux under *P. falcatus* For the sake of clarity of the figure, we omitted to show the δ^13^C values for the control of soluble sugars in tree phloem sap and the 16∶1ω5 NLFA in adhering and bulk soil. The former was −25.53±0.85‰ for *C. macrostachyus* and −25.36±0.33 ‰ for *P. falcatus*, and the latter was −27.7±1.4‰ for *C. macrostachyus* and −29.3±1.1‰ for *P. falcatus.* For leaves and soil CO_2_ efflux data are means ± standard deviation (n = 5). No replicates were taken for phloem sap extraction and soil cores for analysis of 16∶1ω5 NLFA in adhering and bulk soil to keep impact of destructive sampling to the plant-soil system to a minimum.

**Figure 3:**
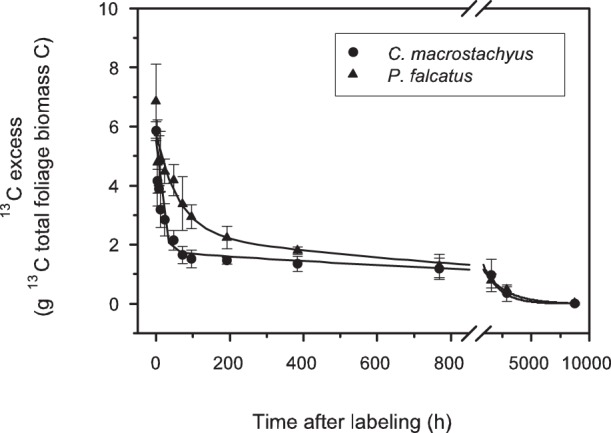
Time course of excess of ^13^C in total foliage biomass of *Croton macrostachyus* and *Podocarpus falcatus* during the one year chasing period. Data are means ± standard deviation (n = 5). Parameters are shown in Table 2.

Before labeling, the experimental and control trees did not differ in the ^13^C natural abundance in the foliage (Fig. 2a, b). Immediately after the chambers were opened (sampling time 0), the δ^13^C of the foliage of both labeled trees was strongly elevated as compared to the control trees, with a δ^13^C value of 1557±871‰ for *C. macrostachyus* and of 248±50.5‰ for *P. falcatus*. The greater net assimilation rate of *C. macrostachyus* was offset by the larger foliar biomass of the *P. falcatus*, thus resulting in only minor differences in the total amount of ^13^C in the foliage of the two species. Excess ^13^C in the foliage, i.e. the ^13^C assimilation calculated with approach 2, was 5.9±0.3 g for *C. macrostachyus* and 6.9±1.3 g for *P. falcatus* (Fig. 3). This result fits well to the ^13^C assimilation as estimated by the decline in the CO_2_ concentration within the chamber, and indicates a pulse labeling efficiency of 61% for *C. macrostachyus* and 59% for *P. falcatus.*

**Table 2:** Results of the fit of exponential functions on the excess of ^13^C in leaves and the cumulative excess of ^13^C in soil CO_2_ efflux related to time after labeling.

	*Croton macrostachyus*				*Podocarpus falcatus*			
Carbon pool	Size	MRT	Half life	*R^2^*	Size	MRT	Half life	*R^2^*
	%	h	h		%	h	h	
Excess of ^13^C in leaves				0.97				0.94
Fast pool	65	14	10		45	55	38	L
Slow pool	30	2000	1386		38	1111	770	
								
Cumulative excess of ^13^C in soil CO_2_ efflux				0.99				0.97
Fast pool	17	417	289		15	2000	1386	
Slow pool	23	10000	6930		†			

Shown are the amount of labeled carbon that was recovered in a given compartment as parameters *a* and *c*, being expressed as the relative size of a fast and a slow pool, the mean residence time (MRT) and the half life of both pools, and the coefficient of determination (*R^2^*). Please note that the size of the pools refer to the percentage of the overall assimilated ^13^C.

† No separation between fast and slow pool could be made.

With time, labeled leaves of both species became ^13^C-depleted, and approached the isotopic signature of the leaves in the reference trees within one year (Fig. 2a, b). The tree species differed in ^13^C recovery kinetics (Fig. 3). In case of *C. macrostachyus* already after 24 hours only about 50% of the total assimilated ^13^C were found in the leaves, whereas for *P. falcatus* foliage a 50% decrease took more than 2 days. The time course of the ^13^C recovery in leaves was best fitted with a double exponential decay function (Table 2), suggesting two pools with different decay constants. The size of the fast pool was larger for *C. macrostachyus* as compared to *P. falcatus*, and the MRT of the label within this pool was almost 4 times shorter.

### ^13^C Translocation Belowground

The soluble phloem sugars of *C. macrostachyus* were mostly mono- and disaccharides with predominance of sucrose (up to 70%). The phloem sap of *P. falcatus* contained in addition the indirect product of photosynthesis d-1-*O*-methyl-*muco*-inositol (OMMI), with a proportion of about 42% of the transported sugars and polyols. The velocity of translocation of recent photoassimilates to the root system via phloem transport differed between the tree species. Soluble sugars in phloem sap of *C. macrostachyus* at 1.3 m stem height were already enriched in ^13^C 4 to 8 hours after labeling, and the tracer peaked in the following 40 hours at 1.3 and 0.5 m height (Fig. 2c). For *P. falcatus* the first evidence of the ^13^C label in phloem soluble sugars appeared 4 hours later, and δ^13^C reached a peak at 1.3 m and 0.5 m height between 72 and 96 hours after the labeling event (Fig. 2d). During the chasing period in both trees the mono- and disaccharides in the phloem sap exponentially became ^13^C depleted (*R^2^*>0.93, *P*<0.0001). In contrast, OMMI in the *P. falcatus* phloem sap showed a relatively constant enrichment of about 2–10‰ above the control throughout the period of sampling (data not shown).

The adhering soil (0–10 cm depth) underneath *C. macrostachuys* had a larger concentration of NLFA 16∶1ω5 than that under *P. falcatus* (18.1±4.6 *versus* 10.4±4.8 nmol g of dry soil^−1^). The same was true for the bulk soils (11.1±1.8 vs 6.1±2.7 nmol g of dry soil^−1^). The enrichment of the AM fungal biomarker with ^13^C appeared synchronized with the transport of the label within the phloem (Fig. 2e, f). Under *C. macrostachyus* the ^13^C in NLFA 16∶1ω5 was elevated already within 48 hours and peaked 4 to 8 days after the labeling. For *P. falcatus* highest ^13^C enrichment of the AM fungal biomarker in adhering soil occurred later (8 to 16 days after labeling) and was less pronounced. The temporal pattern of the tracer in the AM NLFA in bulk soils was similar in shape but the biomarker was less enriched in ^13^C than that in the adhering soils.

### ^13^C Recovery in Soil CO_2_ Efflux

At the beginning of the chasing period the soil CO_2_ efflux rate was 4.8±0.3 µmol m^−2^ s^−1^ under *C. macrostachyus* and 4.6±0.5 µmol m^−2^ s^−1^ under *P. falcatus* (n = 5). With decreasing soil moisture during the first four months after labeling, the soil CO_2_ efflux rates decreased concurrently to 3.1±0.3 µmol m^−2^ s^−1^ under the deciduous tree and to 2.8±0.6 µmol m^−2^ s^−1^ under the conifer.

The temporal course of the tracer’s occurrence in CO_2_ soil efflux mirrored that in the phloem sap and the NLFA 16∶1ω5. Beneath *C. macrostachyus* soil CO_2_ efflux from all 5 collars coincidently showed a remarkable increase in δ^13^C (99.3±4.6‰) already 48 hours after the labeling, reaching a maximum within the next 24 hours (Fig. 2g). Under *P. falcatus* the first evidence of the label in soil CO_2_ efflux occurred around 72 hours after the labeling, and the maximum enrichment (δ^13^C of 3.9±1.4‰) was recorded at day 8 of the chasing period (Fig. 2 h). The time lag between ^13^C photosynthetic uptake and release in soil CO_2_ efflux, as calculated by fitting a quadratic function to the relationships between δ^13^C and the time after labeling [21], were 29 and 51 hours for *C. macrostachyus* and *P. falcatus*, respectively. For both trees, the δ^13^C values of soil CO_2_ efflux decreased exponentially with time (*R* ^2^ = 0.90 for *C. macrostachyus* and 0.86 for *P. falcatus*), being faster *for C. macrostachyus*. At the end of the chasing period the δ^13^C values approached natural abundance under both tree species (Fig. 2g, h).

**Figure 4:**
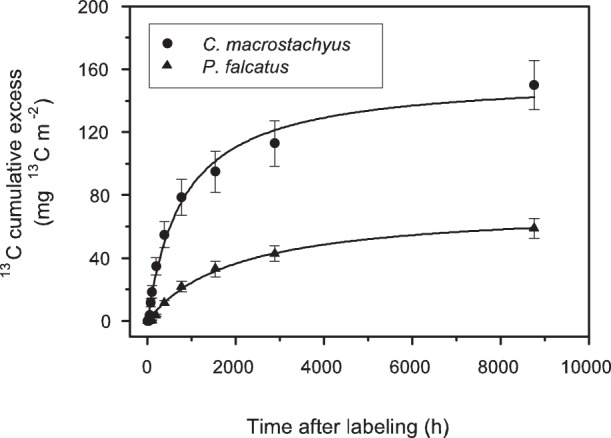
Time course of cumulative excess of ^13^C in soil CO_2_ efflux under C*roton macrostachyus* and *Podocarpus falcatus* during the one year chasing period. Data are means ± standard deviation (n = 5). The curves are fitted with a double exponential function for *C. macrostachyus* and a single exponential function for *P. falcatus*. Parameters are shown in Table 2.

Despite the comparable values of total ^13^C assimilated by the trees during labeling, Fig. 4 shows that the label recovered in the cumulative soil CO_2_ efflux within one year for *P. falcatus* was (14.9±2.4%), which was half that of *C. macrostachyus* (32.2±3.3%). In the case of *C. macrostachyus* the label recovery in soil CO_2_ efflux followed a double-exponential function, indicating that 17% of the overall assimilated carbon was released as fast pool within 17 days (Table 2). In contrast, the kinetics of the label recovery in cumulative soil CO_2_ efflux under *P. falcatus* did not allow one to distinguish between the two sources of soil respiration.

## Discussion

### Labeling

We used two independent approaches to calculate the total amount of assimilated ^13^C by the trees. The first approach was based on the average rate of ^13^CO_2_ uptake as a function of the concentration over the labeling time, and the second estimated the ^13^C excess in leaves immediately after the labeling. In the first approach, uncertainties arose from assumptions in the pressure application for the Ideal Gas Law and the correction of the absorption spectra of the infrared gas analyser, while in the second approach the foliage biomass estimation was quite rough. However, the two independent methods resulted in comparable ^13^CO_2_ uptake by the trees and gave reasonable estimates of the labeling efficiency [70].

The rate of photoassimilation of the early successional *C. macrostachyus* was higher in comparison to the late successional *P. falcatus.* Accordingly, Lüttge *et al.* [49], Fetene and Beck [50], and Seyum *et al*. [51] showed that *C. macrostachyus* had much higher photosynthesis and transpiration rates than *P. falcatus.* This fits well to the general differences in the functional traits of early and late successional, or angio- and gymnosperm tree species [71], [72]. Further, it is widely accepted that species with a longer leaf lifespan (i.e., gymnosperms, like *P. falcatus*) have a lower photosynthetic capacity per mass unit as well as per surface unit [73]. In the following section, we will address how this relates to the carbon allocation belowground.

### Kinetics of Label Recovery and Carbon Allocation

*Croton macrostachyus* lost part of its leaves at the end of the dry season and a flush of new leaves occurred thereafter. However, since most of the label was translocated before the partially shedding of the leaves in February 2009, the effect of this bias on excess of ^13^C in leaves can be considered small. The pattern of the recently fixed ^13^C allocation belowground differed between *C. macrostachyus* and *P. falcatus* in time and magnitude. The gap filling angiosperm had a larger fast pool of the label in foliage which was also having a shorter MRT than the one of the late successional gymnosperm. The MRT of the fast pool of *P. falcatus* was similar as the MRT of 32 hours for the recently fixed carbon in the fast pool of needles of young Scots pine trees, as reported by Högberg *et al*. [10]. A part of the assimilated carbon remained in leaves for longer time, most likely as reserve compounds, such as starch. Generally, leaves with a longer lifespan as those of *P. falcatus* tend to accumulate secondary compounds (i.e., cellulose and phenolics), while those with a shorter lifespan contain larger amounts of proteins [74].

The time lag between the ^13^C uptake and the appearance of ^13^C enriched sugars in phloem sap was about twice as long for *P. falcatus* as for *C. macrostachyus.* The velocity of the phloem transport in the two study trees was approximately 0.4 m h^−1^ (*C. macrostachyus*) and 0.1 m h^−1^ (*P. falcatus*). Due to coarse time resolution of phloem sap sampling (4 hours interval during the first day and then daily for the next 4 days), these values might have some uncertainties, but they are in accordance with other reports of most recently assimilated carbon reaching the phloem within hours to days [10], [13], [75], [76]. The measured carbon phloem transport velocities also agree well with the known differences between deciduous and evergreen species [11], [70], [76]. The different time resolution of belowground transport between gymnosperm *P. falcatus* and angiosperm *C. macrostachyus* could be attributed to the general advances in leaf vein branching, transport of the newly fixed carbon from the sites of CO_2_ fixation to the sieve elements and phloem loading mechanism, developed during the evolution from gymnosperms to angiosperms [42], [43], [70]. The differences in the non-structural carbohydrate composition between the tree species also reflect the evolutionary traits of the phloem with its uniformity in assimilatory metabolites, which are mainly represented by sucrose in angiosperms [42]. In contrast, with OMMI being present in the phloem sap of *P. falcatus,* also other cyclic polyols are ubiquitous in gymnosperms. As they are no direct products of the primary metabolism, they behave more conservatively. Hence, OMNI became less enriched than mono- and disaccharides but contained the label over the whole period of observation.

In line with the different velocities of the ^13^C flux in the phloem sap, the time lag between the ^13^C assimilation by the foliage and the release of the tracer by soil CO_2_ efflux was almost 2 times longer for gymnosperm *P. falcatus* than for angiosperm *C. macrostachyus.* For the angiosperm this result is in accordance with 8-m tall European beech trees, for which a time lag of 2 to 3 days was reported [13]. The gymnosperm values also compare to those of other reports, e.g., Andrews *et al*. [77] identified a time lag of 7 days for a *Pinus taeda* forest labeled in a FACE experiment. Although the total soil CO_2_ efflux rate was comparable throughout the chasing period (see also [54]), the cumulative amount of the label recovered in soil CO_2_ efflux was twice as large for the early successional angiosperm as for the late successional gymnosperm.

In the case of *C. macrostachyus* the temporal resolution of the cumulative label recovered in soil CO_2_ efflux suggests that two different carbon pools with different MRT contributed to soil respiration. A first pool with a MRT of 17 days is closely connected to photosynthesis and reflects the direct use of recent assimilates by the autotrophic continuum. This includes root respiration, transfer to fungal symbionts with subsequent mycorrhizal respiration, and exudation into the rhizosphere, again with subsequent respiration by rhizosphere bacteria [3], [78]. With 17% of the overall assimilated ^13^C, this first pool represented an equal share of the label recovery in the soil CO_2_ efflux as under *P. falcatus* during the whole year of observation. For *P. falcatus* no differentiation of a fast and a slow pool could be calculated. This might be due to the longer storage of assimilates in leaves or metabolites in phloem and their allocation to roots during the whole chasing period. This more conservative flow of photoassimilates to the autotrophic continuum in soil might have contributed to the lack of clear distinction of different pools in soil CO_2_ efflux. Nevertheless it can be assumed also for *P. falcatus* that soil CO_2_ efflux is primarily driven by autotrophic respiration during the first few days [10].

One member of the autotrophic continuum is mycorrhizal fungi. Both tree species are associated with AM, with C. *macrostachyus* is more intensively colonized [79]. This is also shown by larger NLFA 16∶1ω5 concentrations in soil under C. *macrostachyus.* Such larger mycorrhizal colonization has been reported to increase the proportion of plant carbon allocated below ground [80], and to be associated with higher rates of root respiration [81] and soil CO_2_ release [82]. In fact, there was a close temporal coupling between the label peaks of phloem sap and 16∶1ω5 NLFA. This confirms the suggestion of Högberg *et al* [10] of a very pronounced carbon transfer from the plant to the fungal symbiont (though AM in the present case). Hence, a considerable part of the carbon flux in soil occurred through mycorrhiza, which is thought to account for up to one quarter of the carbon assimilated by the tree [43]. Higher rates of respiration by the autotrophic community in AM-colonized plants can be related to increased nutrient uptake. An increased demand for respiratory products (i.e. ATP, NADH) is necessary at each of the four stages of nutrient uptake by an AM-colonized plant, from ion uptake by the external fungal hyphae via ion transport within the fungus, ion export by the internal hyphae to ion uptake by plant root cells [83]. Thus, higher nutrient demand should result in higher carbon investments into the mycelia net, fostering nutrient acquisition. In fact, early successional angiosperms are characterized by much larger nutrient concentrations and turnover rates as late successional gymnosperms [84]. This also corresponds to the higher demand for photoassimilates in case of the former. Accordingly, the earlier and much more pronounced ^13^C enrichment followed by a steeper decline under *C. macrostachyus* than under *P. falcatus* implies the more direct supply of carbon to the fungal symbiont by the early successional tree.

The second pool describes the soil ^13^CO_2_ efflux released by heterotrophic decomposition of structural organic matter [85]. Since leaf litter was collected over the chasing period and did not contribute to the pool of the labeled structural compounds, the heterotrophic efflux must be primarily driven by decomposition of root litter, mycorrhizal hyphae and other rhizobiota. No data are available about the root turnover of the trees under investigation. But assuming that root lifetimes in broadleaf tropical forests (annual precipitation >1000 mm) range from 0.4 to 3.2 years [86], and that the root life span is longer for slow growing than for fast growing tree species [87], we suggest a higher longevity of roots of slow growing *P. falcatus* as compared with the fast growing *C. macrostachyus.* Hence, probably a smaller share of the ^13^C label incorporated into structural components of the root-mycorrhiza system has been mineralized to CO_2_ in case of *P. falcatus*. In total it appears that in case of the late successional *P. falcatus* more of the recently assimilated carbon stays in the plant-soil system, presumably as structural carbon components in above and belowground biomass (Krepkowski, unpublished) and by producing litter with slow decomposition rates [48].

### Conclusions

Our study shows a close temporal coupling with a time lag of one to a few days between assimilation of carbon by the tree canopy and the respiratory activity of the autotrophic continuum in a tropical montane forest, although we caution that the experiment was unreplicated because of expense of whole-tree labeling. Nevertheless, this agrees with recent findings with temperate and boreal trees, suggesting that general mechanisms apply across the biomes.

The rate of carbon allocation into soil is determined by the successional habit of the trees. Corresponding to the functional traits, the early successional angiosperm *C. macrostachyus* pumps carbon much faster and at higher quantities belowground than the late successional gymnosperm *P. falcatus.* The more direct and larger carbon translocation belowground probably reflects the larger energy demand by AM-colonized roots in the belowground autotrophic continuum for an enhanced water and nutrient uptake.

As the continuing degradation of the Afromontane forest leads to the formation of more gaps, the shift in abundance towards gap filling tree species is proceeding. In the long run, the shift in carbon allocation pattern driven by forest degradation may affect the forest carbon balance. Larger carbon allocation of gap filling trees into fast cycling belowground pools likely leads to a decrease of tree biomass carbon accumulation and to declining soil organic carbon contents. We propose this negative effect of early successional angiosperms on ecosystem carbon balance as a worldwide phenomenon which merits further investigation.
